# Satisfaction Levels of Ambulatory Patients with the Quality of Nursing Care: Validation and Application of the Patient Satisfaction with Nursing Care Quality Questionnaire in Albania

**DOI:** 10.3390/nursrep15010004

**Published:** 2024-12-27

**Authors:** Sonila Qirko, Vasilika Prifti, Emirjona Kicaj, Rudina Çerçizaj, Liliana Marcela Rogozea

**Affiliations:** 1 Faculty of Medicine, Transilvania University of Brasov, 500036 Brasov, Romania; vasilika.prifti@unitbv.ro (V.P.); emirjona.kicaj@unitbv.ro (E.K.); rudina.cercizaj@unitbv.ro (R.Ç.); r_liliana@yahoo.com (L.M.R.); 2 Department of Nursing, Faculty of Health, University of Vlora, 9401 Vlora, Albania

**Keywords:** patient satisfaction, ambulatory care, nursing care quality, socio-demographic factors

## Abstract

Background: In the last decades, there has been a growing demand for outpatient services; understanding the factors influencing patient satisfaction is critical for improving healthcare quality. Objectives: This study evaluates patient satisfaction with nursing care and examines how satisfaction varies based on socio-demographic factors in ambulatory settings across five healthcare centers in the municipality of Vlora, Albania. Methods: In this cross-sectional study, a total of 246 patients were surveyed using the Patient Satisfaction with Nursing Care Quality Questionnaire (PSNCQQ), adapted for outpatient contexts, after assessing its validity and reliability. The mean age of the sample was 63.9 ± 13.1 years old with a range of 21 to 94 years, and 47.2% were aged between 50 and 69 years. Results: The results indicate that the overall satisfaction level was fair, with a mean PSNCQQ score of 2.55 on a five-point scale. Socio-demographic factors, such as age, gender, education, and socio-economic status, significantly impacted patient satisfaction. Younger patients (aged 20–49), females, and those with a higher education and socio-economic status reported higher satisfaction. Medical history also played a role, with patients attending general check-ups showing greater satisfaction compared to those with chronic conditions. Older patients tend to report a lower level of satisfaction with the care provided compared to younger patients. Conclusions: Communication and nurse–patient interactions emerged as key areas for improvement, particularly in outpatient settings where care is episodic. These findings suggest that personalized care, improved communication, and greater attention to socio-demographic and medical factors can enhance patient satisfaction in ambulatory care settings.

## 1. Introduction

Over the past four decades, patient satisfaction has been a significant research topic and is expected to intensify with the emphasis on patient-centered care [1]. Extensive literature has reported the factors related to patient satisfaction with care [2]. However, less evidence exists about patient satisfaction in ambulatory settings. Recent trends show the growth of outpatient visits and the use of various levels of training in nursing support roles. Offering healthcare services at the ambulatory level is very critical for healthcare systems. Receiving services from these settings can significantly decrease the cost for healthcare systems as well as improve patient experience [3]. Inadequate nursing care in ambulatory settings can significantly impact patients and healthcare systems. Poor communication, a lack of empathy, or insufficient care plans can lead to misdiagnoses, treatment delays, and increased risks of adverse events, undermining patient safety. Dissatisfaction with care often results in reduced adherence to treatment and follow-up, exacerbating health conditions and increasing the likelihood of hospitalizations or emergency visits [3]. These issues also drive healthcare costs up by negating the cost-saving potential of outpatient services. Psychologically, patients may experience heightened anxiety or stress due to neglect, further impacting their quality of life. Systemically, persistent deficiencies erode trust in healthcare, deterring patients from seeking timely care and hindering preventive efforts. Inadequate care is often rooted in systemic problems such as understaffing and insufficient training, which lead to nurse burnout and high turnover, perpetuating poor-quality care [4]. Addressing these challenges is essential to improving patient satisfaction, outcomes, and the sustainability of outpatient services. Prioritizing high-quality nursing care is crucial for an efficient and equitable healthcare system. As the demand for nurse-led outpatient services grows, it has become increasingly important to understand what factors contribute to patient satisfaction with the quality of nursing care [4]. As nurses continue to expand their role in providing patients with direct care in ambulatory settings, understanding the patient’s view of care has grown in importance [5]. In healthcare systems, patient satisfaction continues to receive considerable attention within the research literature [3]. However, many different terms have been used to describe this concept, such as patient perceptions of care, patient experiences, and patient satisfaction [1]. The satisfaction level of a patient’s experiences is based on their nursing care [5]. It has been suggested that it makes sense to assume that satisfaction with nursing care is important overall, given the significant amount of time patients spend interacting with nurses [6]. Furthermore, patient and nurse satisfaction within ambulatory settings is worthy of investigation, and some have suggested that satisfaction is elevated within outpatient settings compared to when care is provided within inpatient settings [2,7]. While enhancing the satisfaction of patients and their families is intrinsically valuable, there is evidence to suggest that a positive patient experience in outpatient medical facilities can lead to more suitable healthcare utilization and better overall health results [8]. Research on patient satisfaction has expanded rapidly, but the field faces several issues. These include inconsistent findings and poor research practices, such as unclear definitions and methods. Additionally, there is often not enough focus on the complex interactions between healthcare quality and patients’ social and health factors [9]. Continuing to evaluate and improve patient satisfaction with nursing care highlights the need for ongoing theory development within the field, which integrates the many social, organizational, and clinical processes, structures, and outcomes that influence the quality of patients’ encounters with nurses [2]. To establish trust between the patient and the nurse, a concrete and clear healthcare plan, good communication, and empathy are needed. Increased satisfaction positively affects the quality of nursing care and the well-being of patients [4,10]. Patient satisfaction with nursing care has been a prominent focus of research for decades, emphasizing its critical role in healthcare quality and outcomes. Existing studies have extensively explored satisfaction factors in inpatient settings, but there is limited evidence on patient satisfaction specific to ambulatory care. Furthermore, the transient and episodic nature of outpatient interactions presents unique challenges that differ from the continuity of inpatient care. While previous research highlights the importance of communication, empathy, and tailored care strategies, inconsistencies in definitions, methodologies, and findings complicate the field. Our study builds on these insights by focusing on socio-demographic influences on satisfaction within ambulatory care in Albania, a context that remains underrepresented in the global literature. By addressing these gaps, this research contributes original value, providing localized evidence to inform nursing practices and enhance patient satisfaction in outpatient settings. The primary purpose of this study was to assess patient satisfaction with the quality of nursing care in ambulatory settings across five healthcare centers in Vlora, Albania. Additionally, the study aimed to validate the Albanian version of the Patient Satisfaction with Nursing Care Quality Questionnaire (PSNCQQ) to ensure its suitability and reliability in measuring patient satisfaction within this specific context. Our key research questions focus on the satisfaction level of ambulatory patients about the quality of nursing care as well as on the impact of different factors.

## 2. Materials and Methods

### 2.1. Study Design

A cross-sectional study was conducted to evaluate the satisfaction levels of ambulatory patients regarding the quality of nursing care. The research involved the validation and application of the Patient Satisfaction with Nursing Care Quality Questionnaire (PSNCQQ) in the context of healthcare services in Albania, aiming to provide insights into patient perspectives and improve nursing care standards.

### 2.2. Study Setting

The study was conducted in five public urban community health centers (HCs) located in Vlora, Albania, over a three-month period from April to June 2024. These centers serve an urban population of approximately 144,000 inhabitants, providing a vital source of healthcare for the city’s residents. As primary healthcare providers, these centers focus on a wide range of services, including preventive care, chronic disease management, maternal and child health, and treatment of minor emergencies.

The urban population served by these health centers is diverse, with a mix of socio-economic backgrounds. These health centers play a critical role in addressing the health needs of the community by offering accessible and affordable care. The significant patient flow and dense urban population make these centers well-suited for evaluating nursing care quality and its influence on patient satisfaction. This setting offers valuable insights into nursing practices and patient experiences within Albania’s urban healthcare framework.

### 2.3. Participants

A convenience sampling technique was employed to recruit patients who received ambulatory care at five HCs in the Vlora district. The inclusion criteria for this study required participants to be 18 years of age or older, able to speak and understand Albanian, cognitively alert, physically capable of responding to survey questions, and willing to participate. Exclusion criteria were also applied, including individuals who were critically ill, unable to provide informed consent, or had significant communication barriers such as language barriers other than Albanian or hearing impairments without support.

Data were collected by four trained researchers through face-to-face interviews after the visit. An interview lasted approximately thirteen minutes.

Patients who agreed to participate in the study were given a detailed explanation of its purpose and signed informed consent forms. Those who declined to participate cited reasons such as a lack of time or disinterest. Surveys that were incomplete were excluded from the study.

### 2.4. Variables

Data collection was conducted using a personal information form and the Patient Satisfaction with Nursing Care Quality Questionnaire (PSNCQQ). The study examined a variety of variables to understand the factors influencing patient satisfaction with nursing care in ambulatory settings. Among the demographic variables, age was categorized into three groups: younger adults (20–49 years), middle-aged individuals (50–69 years), and older adults (70 years and above). Gender was recorded as male or female, and education levels were classified as secondary, high school, university, or postgraduate. Marital status included categories such as cohabiting, single, divorced, married, or widowed. Socio-economic status was assessed based on data from the Institute of Statistics (INSTAT). Low income is defined as ≤40,000 Albanian Lek (ALL) per month, moderate income as 40,001 to 75,114 ALL per month, and high income as ≥75,115 ALL per month [11]. Employment status was also recorded, with participants identified as unemployed, employed, self-employed, or retired. In terms of medical history, the study explored the reasons for patient visits. These reasons ranged from general health check-ups to consultations for specific conditions, including cardiovascular, respiratory, gastrointestinal, musculoskeletal, or other illnesses.

### 2.5. Data Sources/Measurement

#### 2.5.1. Instrument Used Patient Satisfaction with Nursing Care Quality Questionnaire—PSNCQQ

The Patient Satisfaction with Nursing Care Quality Questionnaire (PSNCQQ) is a tool designed to assess patients’ perceptions of the quality of nursing care they receive during hospital stays. It was developed in 2005 by Dr. Heather K. Spence Laschinger and her colleagues at Western University in Canada [12]. The PSNCQQ consists of 19 items, each rated on a 5-point Likert scale ranging from 1 (poor) to 5 (excellent). Higher scores indicate greater satisfaction with nursing care quality.

The PSNCQQ is designed to evaluate the degree of anticipated needs, gauge patient satisfaction after a short-term hospitalization, and examine the impact of socio-demographic, personal, and other factors at a basic level.

Apart from the socio-demographic data, the scale consists of 19 items related to various aspects of nursing activities, including nurses’ attention, kindness, respect, courtesy, skills, competence, and the fulfilment of patient needs.

The PSNCQQ was adapted to be used in the context of outpatient (ambulatory) patients. For this purpose, questions related to the hospital environment were removed from the questionnaire, specifically: question 5, “Informing Family or Friends: How well the nurses kept them informed about your condition and needs”, as well as questions 17 and 18, which are, respectively, “Discharge Instructions: how clearly and completely the nurses told you what to do and what to expect when you left the hospital” and “Coordination of Care after Discharge: Nurses’ efforts to provide for your needs after you left the hospital”.

From the section on overall perception, only the question related to patients’ opinion about their health was retained: “In general, would you say your health is…” A 5-point Likert-type scale is used, scored between “(5) excellent” and “(1) poor”.

#### 2.5.2. Psychometric Properties of the PSNCQQ

1. Validity and reliability analysis

For the 16 questions in the questionnaire, an analysis of validity, reliability, and factor analysis was carried out to determine the structure of the questionnaire.

2. Translation and Content Validity

Initially, forward–backward translation was performed. The PSNCQQ was translated into Albanian, and both the linguistic and conceptual equivalence of the items were confirmed. A back-translation process was carried out to ensure consistency between the English and Albanian versions of the scale. The original scale was translated into Albanian by translators highly proficient in both languages. A group of five bilingual experts, including a doctor, two nursing faculty lecturers, a nurse manager, and a linguist, analyzed the expressions used in the scale. Each expression was reviewed both individually and in combination, leading to the selection of the most appropriate wording for the 16 items.

The Content Validity Index (CVI) was used to estimate the validity of the items. The backward translation from Albanian to English was conducted by two trained linguists (English teachers) with expertise in both languages. The backward-translated version and the original version of the PSNCQQ were compared and found to be highly consistent in meaning, with adjustments made to align with the specific characteristics of the country. Subsequently, the expert panel convened to evaluate the validity of the scale. Five experts, including nursing academicians specializing in medical nursing and nursing administration, reviewed and provided their opinions on the adequacy of meaning and content. A pilot study was conducted from 30 October to 30 November 2024, involving 50 patients to identify any unclear questions in the scale. The data collected from this pilot study were excluded from the final data analysis. Based on the results of the pilot study, minor adjustments were made to the wording of certain items to enhance their clarity.

3. Construct Validity

Factor analysis of the Albanian version of the scale yielded a single-factor structure, with factor loadings ranging from 0.732 to 0.942.

4. Internal Consistency

In this study, the correlation coefficients between the average PSNCQQ item scores ranged from 0.71 to 0.93, indicating a satisfactory level of reliability. Cronbach’s α for the PSNCQQ, calculated to assess internal consistency and uniformity, was 0.885, which is considered very high.

5. Test-Retest Reliability

As a part of the pilot study, the test-retest reliability was evaluated by administering the same questionnaire to a sample of 50 patients two weeks apart. The results were consistent with those reported by Laschinger et al., confirming the stability and reliability of the instrument over time [12]. As a result, the Albanian version of the PSNCQQ can be regarded as having excellent psychometric properties, closely aligning with those of the original scale.

### 2.6. Bias

The study implemented several measures to address potential sources of bias. Clear inclusion and exclusion criteria ensured a relevant and capable sample, while the PSNCQQ underwent rigorous adaptation and validation, including forward-backward translation, expert review, and pilot testing, to ensure cultural and contextual suitability. Data collection was standardized through face-to-face interviews conducted by trained researchers, minimizing variability. A pilot study identified and corrected ambiguities in the questionnaire, improving clarity and reducing response bias. Ethical guidelines, including informed consent, were strictly followed to ensure voluntary and unbiased participation. Finally, appropriate statistical methods were employed to analyze the data, ensuring robust and reliable findings.

### 2.7. Study Size

The sample size for the study was determined based on a review of the relevant literature, aiming to detect a 25% satisfaction rate among ambulatory patients. The calculation utilized a single proportion formula with z = 1.96 for a 95% confidence interval (CI) and a 5% margin of error [13]. The study included 246 ambulatory patients, achieving a response rate of 85.4%.

### 2.8. Quantitative Variables

Age was measured in years and categorized into three groups: 20–49, 50–69, and ≥70, allowing for the analysis of satisfaction trends across age groups. PSNCQQ scores were assessed on a 5-point Likert scale (1 = poor, 5 = excellent), evaluating various aspects of nursing care. An overall mean satisfaction score was calculated to summarize participants’ general satisfaction levels.

### 2.9. Statistical Methods

Data were analyzed using SPSS software (IBM Corp. Released 2012. IBM SPSS Statistics for Windows, Version 25.0; IBM Corp., Armonk, NY, USA). Descriptive statistics, including frequencies, percentages, means, and standard deviations, were used to summarize the socio-demographic characteristics of the study participants, as well as their responses to the Patient Satisfaction with Nursing Care Quality Questionnaire (PSNCQQ). Continuous variables, such as age and PSNCQQ scores, were presented as means with standard deviations, while categorical variables, including gender, marital status, education, and socio-economic status, were presented as frequencies and percentages. The data were normally distributed and, therefore, parametric tests were used.

To examine the relationships between socio-demographic characteristics and patient satisfaction, several inferential statistical analyses were conducted. Independent sample *t*-tests were used to compare the mean satisfaction scores between two groups (e.g., male vs. female participants). For comparisons involving more than two groups, such as age categories or education levels, one-way analysis of variance (ANOVA) was used to determine whether there were statistically significant differences in satisfaction scores.

Post hoc comparisons using the Bonferroni correction were applied to identify specific group differences when significant results were observed in the ANOVA tests. The overall satisfaction with care measure was dichotomized into 2 groups: excellent/very good and poor/fair responses, and univariate and multivariate logistic regression was used to analyze the relationship between socio-demographic factors and the PSNCQQ score. For all statistical tests, a *p*-value of less than 0.05 was considered statistically significant.

### 2.10. Ethical Considerations

All ethical guidelines as well as the Helsinki declaration were strictly followed for this study. Permission was obtained from the instrument’s developer. Before data collection, the research protocol was reviewed and approved by the appropriate scientific ethics committee of the University “Ismail Qemali” No. 113/1; written approval to conduct the research was also obtained from local health authorities in the Vlora municipality. Participation was voluntary, and patients provided written consent with the option to withdraw at any time.

## 3. Results

**Figure 1:**
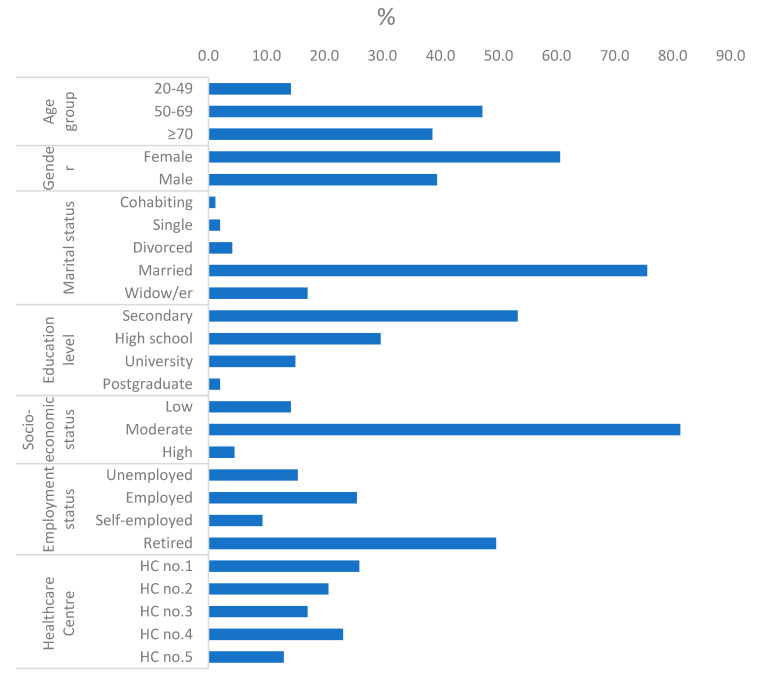
Patient characteristics (N = 246). HC: health center.

Socio-demographic characteristics of the participants (N = 246) are presented in Figure 1. The mean age of the sample was 63.9 years old (SD 13.1) with a range of 21 to 94 years, and 47.2% were aged between 50 and 69 years. A total of 60.6% of the participants were of female gender, married (75.6%), had a secondary education level (53.3%), reported a moderate socio-economic level (81%), and retired (49.6%).

### 3.1. PSNCQQ Scores

Overall, patients’ PSNCQQ scores ranged between 1 and 5, with an average score of 2.55 (SD 0.93). This indicated that the level of satisfaction with nursing care was fair. All items of the questionnaire present an average, almost uniform level in satisfaction with the care (Table 1).

The analysis of PSNCQQ scores for the perception-related item “In general, would you say your health is” showed that 35.8% responded “well” and 34.1% responded “moderate”.

### 3.2. Comparison of PSNCQQ Scores According to Patients’ Socio-Demographic Characteristics

The mean PSNCQQ score of patients in the age group 20–49 years was significantly higher, at 3.19 (SD 1.14), in relation to those observed for patients aged 50–69 years, 2.45 (SD 0.89), and aged ≥70 years, 2.43 (SD 0.81), (*p* < 0.01). Females had a higher score, 2.64 (SD 0.98), as compared to males, 2.40 (SD 0.84), with a significant difference (*p* = 0.044).

The widowed patients’ mean PSNCQQ score was found to be statistically higher (1.81 SD 0.75) than that of the married patients’ (1.57 SD 0.62), and the difference was significant (*p* < 0.01).

Postgraduates, patients with high socio-economic status, and employed individuals had significantly higher scores compared to those with lower education, socio-economic status, or employment status. Additionally, patients attending HC No. 1 scored significantly higher than those at other centers (Table 2).

### 3.3. Comparison of PSNCQQ Scores According to Patients’ Medical Histories

Only the patients who were presented for a general check-up had a significantly higher score of 3.75 (SD 1.20) compared to patients that were presented to consult for their disease (*p* = 0.003). All patients referred a fair level of satisfaction with the nursing care varying from 2.39 (SD 1.12) to 2.85 (SD 0.88) (Table 3).

Overall, 64 (26%) (95% CI 20.63 to 31.95) of patients were satisfied with the quality of care provided by nurses.

In the univariate analysis, increasing age is associated with a decrease in satisfaction, and males have a lower level of satisfaction compared to females (Table 4). Patients with a university or postgraduate education level, those with a high socio-economic status, and employed or self-employed patients are more likely to be satisfied with the quality of nursing care.

In the multivariate analysis, the determinants of patient satisfaction with the quality of nursing care were found to be university or postgraduate education and having a high socio-economic status.

## 4. Discussion

In this study, patient satisfaction was evaluated from various aspects of their experiences. The study aims to assess patient satisfaction to ensure that nursing care is grounded in evidence-based practices and to enhance the overall quality and safety of care provided.

The validation of the Patient Satisfaction with Nursing Care Quality Questionnaire (PSNCQQ) demonstrates its reliability and suitability for assessing nursing care satisfaction in ambulatory settings in Albania. The rigorous adaptation process included forward-backward translation for linguistic and conceptual equivalence and review by a multidisciplinary expert panel to ensure content validity. Pilot testing with 50 patients refined the questionnaire, enhancing clarity and usability. Factor analysis confirmed a single-factor structure with high factor loadings, while a Cronbach’s α of 0.885 and test-retest reliability affirmed its consistency and stability. These findings establish the PSNCQQ as a reliable and valid tool for evaluating patient satisfaction.

This study offers significant insights into the satisfaction levels of ambulatory patients regarding the quality of nursing care in five health centers in Vlora, Albania. While the overall satisfaction was rated as fair, with an average PSNCQQ score of 2.55 (on a 1–5 scale), the results reveal important influences such as socio-demographic characteristics, medical history, and communication quality that play pivotal roles in shaping patient satisfaction. These findings corroborate broader trends in the healthcare literature, which underscore the complexity of measuring patient satisfaction, particularly in outpatient settings where interactions with healthcare providers are more transient and episodic compared to inpatient settings [14,15].

### 4.1. Socio-Demographic Factors and Satisfaction

The significant variations in satisfaction levels across different socio-demographic factors suggest that patient diversity needs to be a focal point in ambulatory care. Younger patients (aged 20–49) reported significantly higher satisfaction (mean = 3.19) compared to older patients (50–69 and 70+) and in the univariate analysis, increasing age is associated with a decrease in satisfaction, which aligns with previous findings showing that younger individuals often have more favorable experiences in healthcare settings due to different expectations or greater familiarity with technology-enhanced care systems [2,16]. Older adults, on the other hand, may face increased complexities in their care needs, contributing to lower satisfaction scores. Additionally, older patients typically present with more chronic conditions and require more comprehensive care management, which can affect their satisfaction levels [2,9]. Studies indicate that older individuals often report lower satisfaction when their healthcare needs are more complex, which may be due to perceived gaps in communication, care coordination, or unmet expectations regarding the thoroughness of care [4]. This may explain the lower satisfaction scores among older patients in our study, as they likely experience more challenges in navigating their care. These results reinforce the importance of age-appropriate communication strategies and interventions in outpatient settings [17].

Previous research suggests that age plays a crucial role in shaping patients’ expectations and perceptions of nursing care quality; [18] found that younger patients tend to report higher levels of satisfaction with healthcare services, potentially due to greater familiarity with modern, technology-enhanced care systems. This finding supports our results, where younger patients reported higher satisfaction scores. Moreover, younger patients visit healthcare services less and may also have more optimistic expectations of healthcare interactions, leading to higher ratings of satisfaction.

Researchers [4,15] have shown that younger patients often feel more engaged in their care, which can enhance their overall experience and satisfaction. They tend to have higher expectations for communication, which, when met, significantly boosts their satisfaction levels. On the contrary, older patients may encounter communication barriers or have more complex healthcare needs, making their care experience less straightforward and potentially leading to lower satisfaction scores.

However, not all studies have found a negative correlation between age and patient satisfaction. An older study [19] has suggested that older patients may be more satisfied due to lower expectations or a higher level of trust in healthcare providers. This discrepancy in findings highlights the variability in patient expectations across different healthcare settings and populations. For instance, older patients in more stable health conditions may report higher satisfaction compared to those with multiple comorbidities requiring frequent and complex interventions.

Gender also played a notable role, with females reporting higher satisfaction levels than males. This finding is consistent with previous studies that suggest women tend to be more engaged in their healthcare, are more likely to communicate their concerns, and generally have higher expectations regarding the interpersonal aspects of care [1,20]. Given that communication and empathy are often key components of patient satisfaction, it is unsurprising that female patients scored higher in satisfaction, reflecting a broader trend in the healthcare literature where women tend to report higher satisfaction across a range of services [5].

Education and socio-economic status also influenced satisfaction scores. Patients with higher education levels, particularly postgraduates, reported significantly higher satisfaction. Educated patients may have greater health literacy, which allows them to better understand and engage with their care, thus leading to more favorable care experiences [21]. Similarly, patients from higher socio-economic backgrounds reported higher satisfaction (mean = 3.33), likely due to their enhanced access to healthcare resources, which can afford them more autonomy and choice in their healthcare options [4,14].

### 4.2. Medical History and Satisfaction

The influence of medical history on satisfaction was also noteworthy. Patients who presented for general check-ups reported the highest satisfaction scores (mean = 3.75), significantly higher than patients seeking care for chronic conditions such as cardiovascular or respiratory diseases. This reflects findings from prior studies which show that patients with less complex health issues, such as those attending for routine health check-ups, tend to have more straightforward and positive care experiences compared to those with chronic or debilitating illnesses [9,22]. Patients managing chronic diseases may have higher expectations for care coordination and thoroughness, and any perceived gaps in care delivery could significantly impact their satisfaction [23].

For patients with health conditions such as musculoskeletal or gastrointestinal diseases, satisfaction levels were comparatively higher than for those with cardiovascular or respiratory issues, though these results need to be carefully interpreted due to the higher number of patients with cardiovascular or respiratory issues. This finding suggests that the complexity of care needs influences patient satisfaction, and it underscores the need for tailored nursing strategies to better meet the expectations of patients with more intricate health conditions [3]. These patients likely require more comprehensive care and clear communication about their long-term management plans [21], which highlights the importance of personalized care pathways for different medical histories [24].

### 4.3. Communication and Its Impact on Satisfaction

The quality of communication between nurses and patients was one of the lower-rated aspects of care in this study, with patients reporting lower satisfaction in areas such as the willingness of nurses to answer their questions. This mirrors previous research that highlights communication as a key determinant of patient satisfaction, particularly in outpatient settings where patients often have limited time with healthcare providers [5,25]. Effective communication is essential not only for improving satisfaction but also for enhancing patients’ understanding of their health conditions and treatments, which can lead to better adherence to care plans [5].

Communication is also closely tied to patients’ perceptions of empathy and attentiveness from healthcare providers. In ambulatory settings, where nurse–patient interactions are often brief, the quality of these interactions becomes even more important [26]. Training nurses to improve their communication skills—particularly in explaining medical information in a way that is easily understood—can lead to significant improvements in patient satisfaction. This is supported by Papp et al. [27], who found that improving nurse–patient communication can enhance overall satisfaction and lead to better health outcomes, especially in outpatient care where continuity of care is more fragmented.

### 4.4. Implications for Nursing Practice and Policy

The findings of this study have important implications for nursing practice, particularly in ambulatory care settings. First, it is evident that communication remains a critical area for improvement. Nurses should be equipped with the necessary skills to effectively communicate with patients, especially in short, high-impact encounters typical of outpatient care [28]. Enhanced communication training that emphasizes clear, concise, and empathetic interactions with patients can lead to better patient outcomes and satisfaction [29].

In addition, healthcare providers should consider the diverse needs of different patient groups when developing care strategies. Older patients and those with chronic conditions in particular, may require more personalized care plans and additional support to navigate their healthcare options. Addressing these needs could improve satisfaction levels for these more vulnerable patient populations [13,30].

Policymakers should also focus on improving the coordination of care in ambulatory settings. The fragmented nature of outpatient care can result in patients receiving disjointed information from various providers, which can negatively impact their satisfaction [24]. By fostering a more integrated approach to care delivery, particularly for patients with chronic conditions, healthcare systems can improve the continuity and quality of care, thus enhancing patient satisfaction [31].

### 4.5. Strength and Limitations

The strengths of this study include being the first of its kind conducted in Albania, involving a representative number of healthcare centers. The response rate was high, and the study demonstrated good results for validity and reliability, adding to its credibility and potential impact.

The limitations of this study include its cross-sectional design, it was conducted in only one city, making it difficult to generalize the results to a broader population, especially since rural areas were not included.

## 5. Conclusions

This study offers valuable insights into the levels of satisfaction among ambulatory patients regarding the quality of nursing care in Vlora, Albania, by applying a validated and adapted version of the PSNCQQ. The findings underscore that patient satisfaction in outpatient settings is influenced by a range of factors, including socio-demographic characteristics, medical history, and the quality of nurse–patient communication. Overall, the results indicate that while patient satisfaction levels were fair, significant opportunities exist for improvement, particularly in communication, care coordination, and personalized nursing care. Satisfaction levels were significantly higher among younger patients, females, those with higher education levels, and individuals with a higher socio-economic status. This highlights the need for tailored approaches that address the diverse expectations and needs of patients based on their age, gender, education, and economic background. Patients attending general check-ups reported higher satisfaction compared to those managing chronic or complex conditions. This disparity underscores the importance of prioritizing care coordination, clear communication, and personalized care plans for patients with chronic illnesses. Nurse–patient interactions were identified as a critical determinant of patient satisfaction. Specific areas requiring attention include the clarity of explanations, responsiveness to patient concerns, and the ability to establish trust and empathy during brief outpatient encounters. Improving patient satisfaction with nursing care in outpatient settings requires a multifaceted approach that addresses critical areas of nursing practice and healthcare delivery. First, healthcare providers should prioritize enhanced training for nurses, equipping them with advanced communication skills. Clear, empathetic, and patient-centered interactions are essential to building trust, understanding patient needs, and delivering care that aligns with their expectations.

Second, personalized care strategies should be developed and implemented. These strategies must cater to the unique needs of specific patient groups, such as older adults and those with chronic conditions. Tailored interventions can help address the complexities of these patients’ care requirements, ensuring they feel supported and valued.

Finally, improving care coordination is crucial. Healthcare providers should work to establish integrated care delivery systems that streamline patient experiences. By minimizing the fragmentation often associated with outpatient care, these systems can create a seamless journey for patients across various touchpoints in the healthcare process, ultimately enhancing their satisfaction and overall outcomes.

This study reinforces the critical role of socio-demographic characteristics, communication, and care complexity in shaping patient satisfaction with nursing care. Addressing these factors through targeted strategies can enhance the quality of nursing care and improve patient outcomes in ambulatory settings. By prioritizing personalized care, effective communication, and care coordination, healthcare systems in Albania can achieve more patient-centered and equitable service delivery.

## Figures and Tables

**Table 1:** Distribution of patient satisfaction with nursing care quality questionnaire (PSNCQQ) scores (N = 246).

Items	Mean	SD ^†1^	Min ^†2^	Max ^†3^
1. Information You Were Given: how clear and complete the nurses’ explanations were about tests, treatments, and what to expect.	2.65	0.99	1	5
2. Instructions: how well nurses explained how to prepare for tests and examinations.	2.58	1.01	1	5
3. Ease of Getting Information: willingness of nurses to answer your questions.	2.45	1.02	1	5
4. Information Given by Nurses: how well nurses communicated with patients, families, and doctors.	2.53	1.06	1	5
5. Involving Family or Friends in Your Care: how much they were allowed to help in your care.	2.74	1.28	1	5
6. Concern and Caring by Nurses: courtesy and respect you were given; friendliness and kindness.	2.69	1.05	1	5
7. Attention of Nurses to Your Condition: how often nurses checked on you and how well they kept track of how you were doing.	2.39	1.15	1	5
8. Recognition of Your Opinions: how much nurses ask you what you think is important and give you choices.	2.25	1.15	1	5
9. Consideration of Your Needs: willingness of the nurses to be flexible in meeting your needs.	2.54	1.08	1	5
10. The Daily Routine of the Nurses: how well they adjusted their schedules to your needs.	2.28	1.10	1	5
11. Helpfulness: ability of the nurses to make you comfortable and reassure you.	2.54	1.10	1	5
12. Nursing Staff Response to Your Calls: how quick they were to help.	2.57	1.11	1	5
13. Skill and Competence of Nurses: how well things were done, like giving medicine and handling IVs.	2.71	1.14	1	5
14. Coordination of Care: the teamwork between nurses and other staff who took care of you.	2.60	1.09	1	5
15. Restful Atmosphere Provided by Nurses: amount of peace and quiet.	2.63	1.17	1	5
16. Privacy: provisions for your privacy by nurses.	2.64	1.11	1	5
Average PSNCQQ score	2.55	0.93	1	5

^†1^ SD, standard deviation; ^†2^ min, minimum; ^†3^ max, maximum.

**Table 2:** Comparison of patient satisfaction with nursing care quality questionnaire scores based on patients’ socio-demographic characteristics (N = 246).

Variables	N	Mean ± SD	t/F	*p*-Value
Age group			10.058	<0.001
20–49	35	3.19 ± 1.14		
50–69	116	2.45 ± 0.89		
≥70	95	2.43 ± 0.81		
Gender			2.030	0.044
Female	149	2.64 ± 0.98		
Male	97	2.40 ± 0.84		
Marital status			1.828	0.124
Cohabiting	3	3.71 ± 1.45		
Single	5	2.41 ± 0.67		
Divorced	10	2.16 ± 0.99		
Married	186	2.53 ± 0.94		
Widow/er	42	2.68 ± 0.84		
Education level			9.225	<0.001
Secondary	131	2.39 ± 0.81		
High school	73	2.48 ± 0.95		
University	37	3.11 ± 1.04		
Postgraduate	5	3.72 ± 0.95		
Socio-economic status			6.804	0.001
Low	35	2.18 ± 0.80		
Moderate	200	2.57 ± 0.92		
High	11	3.33 ± 1.05		
Employment status			6.077	0.001
Unemployed	38	2.32 ± 0.97		
Employed	63	2.97 ± 0.98		
Self-employed	23	2.50 ± 1.01		
Retired	122	2.41 ± 0.82		
Health Centre			7.535	<0.001
HC no.1	64	2.99 ± 0.95		
HC no.2	51	2.54 ± 0.77		
HC no.3	42	2.54 ± 1.00		
HC no.4	57	2.11 ± 0.85		
HC no.5	32	2.46 ± 0.83		

HC: health center.

**Table 3:** Comparison of patient satisfaction with nursing care quality questionnaire scores according to patients’ medical histories (N = 246).

Medical History	N	Mean	SD
Check Up	6	3.75	1.20
Cardiovascular and Metabolic Diseases	154	2.42	0.89
Respiratory Diseases	19	2.39	1.12
Gastrointestinal and Renal Diseases	34	2.85	0.88
Musculoskeletal and Rheumatic Diseases	22	2.76	0.89
Other	11	2.60	0.66

**Table 4:** Univariate and multivariate logistic regression for determinants of outpatient satisfaction.

Variables	Univariate		*p*	Multivariate		*p*
	OR	95% CI		aOR	95% CI	
Age (years)	0.96	0.94; 0.99	0.007	0.98	0.94; 1.02	0.442
Gender						
Female	Ref.			Ref.		
Male	0.47	0.25; 0.88	0.020	0.68	0.33; 1.37	0.283
Marital status						
Single	Ref.			Ref.		
Married/Cohabiting	1.40	0.15; 12.8	0.765	3.59	0.34; 37.2	0.283
Divorced/Widow	1.87	0.19; 18.2	0.588	9.20	0.76; 110.2	0.080
Education level						
Secondary	Ref.			Ref.		
High school	1.53	0.76; 3.09	0.231	1.18	0.55; 2.53	0.658
University/Postgraduate	5.36	2.51; 11.45	<0.0001	3.19	1.21; 8.36	0.018
Socio-economic status						
Low	Ref.			Ref.		
Moderate	1.97	0.72; 5.40	0.184	1.80	0.56; 5.75	0.316
High	9.45	1.99; 44.77	0.005	5.87	0.02; 1.01	0.050
Employment status						
Unemployed	Ref.			Ref.		
Employed/Self-employed	3.33	1.25; 8.64	0.016	1.76	0.59; 5.22	0.305
Retired	1.30	0.48; 3.49	0.597	1.13	0.31; 4.04	0.850

## Data Availability

The raw data supporting the conclusions of this article will be made available by the authors on request.

## Public Involvement Statement

There was no public involvement in any aspect of this research.

## Guidelines and Standards Statement

This manuscript was drafted against the STROBE guidelines for observational research. A complete list of reporting guidelines can be accessed via the equator network: https://www.strobe-statement.org/ (accessed on 6 November 2024).
